# Comparative Genomics of the Tea *XTH* Gene Family Reveals the Involvement of *CsXTH27* in Internode Elongation

**DOI:** 10.3390/ijms27167388

**Published:** 2026-08-18

**Authors:** Hong Shen, Fengshui Yang, Liping Zhang, Aihua Zhu, Lan Zhang, Xin Li, Peiqiang Wang, Shuixing Zhu

**Affiliations:** 1 College of Horticulture, Qingdao Agricultural University, Qingdao 266109, China; 2 State Key Laboratory of Tea Plant Germplasm Innovation and Resource Utilization, Tea Research Institute, Chinese Academy of Agricultural Sciences, Hangzhou 310008, Chinalixin@tricaas.com (X.L.); 3 College of Food Science, Southwest University, Chongqing 400715, China; 4 Agricultural Technology Promotion Center in Xianju County, Taizhou 317399, China

**Keywords:** *XTH* gene family, *Camellia sinensis*, comparative genomics, internode elongation, *CsXTH27*

## Abstract

Xyloglucan endotransglucosylase/hydrolases (XTHs) are key cell-wall-remodeling enzymes that mediate xyloglucan modification, cell expansion, and organ elongation, yet the composition and variation in the *XTH* gene family across tea accessions remain unclear. In this study, we systematically identified and comparatively analyzed the *XTH* gene family across the genomes of nine tea accessions, yielding 477 putative *XTH* genes. Phylogenetic, conserved-motif, protein-domain, and conserved catalytic-motif analyses indicated that the major structural features of tea XTH proteins are broadly conserved, whereas orthogroup-based presence–absence variation (PAV) analysis revealed core, variable, and accession-specific components within the family. Using transcriptomic expression profiles across eight tissues, we selected eight *CsXTH* genes for internode expression analysis. Reverse transcription quantitative PCR (RT-qPCR) showed that all eight genes were expressed at significantly higher levels in the third internode (I3) than in the second internode (I2) in ‘Longjing 43’ (LJ43), and *CsXTH27* retained the same internode-associated expression pattern in ‘Baihaozao’ (BHZ) and ‘Zhongcha 108’ (ZC108), supporting its selection for functional analysis. Transient overexpression of *CsXTH27* significantly increased the lengths of I2, I3, and I4, as well as the total length of I1–I4, compared with the empty-vector control. This phenotype was accompanied by significantly increased XTH protein content in stems and an apparent tendency toward larger pith parenchyma cell profiles. Overall, these findings reveal both structural conservation and accession-level variation in the tea *XTH* gene family and provide preliminary functional evidence linking *CsXTH27* to shoot internode elongation.

## 1. Introduction

Tea plant (*Camellia sinensis* (L.) O. Kuntze) is an economically important perennial crop, and its young shoots are the primary harvested organs for tea production. Internode length is a key architectural trait of young shoots that influences shoot form, fresh leaf yield, and suitability for mechanical harvesting [1,2,3,4]. With the increasing adoption of mechanical tea harvesting, internode length has become increasingly important for determining cutting positions, maintaining bud-and-leaf integrity, and improving overall harvesting quality [1,3]. At the developmental level, internode elongation is closely associated with cell elongation, a process largely constrained by cell wall extensibility and dynamic remodeling [5,6]. Genes involved in cell wall remodeling may therefore provide a molecular link between cell-wall dynamics and internode elongation.

Cell wall remodeling is a fundamental process underlying plant organ growth and development [6]. Xyloglucan, a major hemicellulosic component of the primary cell wall, interacts with cellulose microfibrils and contributes to the structural framework and mechanical properties of the cell wall. Dynamic rearrangement of the xyloglucan network affects cell wall extensibility and thereby influences cell elongation [5,7,8,9,10]. The *XTH* gene family encodes xyloglucan endotransglucosylase/hydrolase proteins that catalyze the cleavage and religation of xyloglucan chains and, in some family members, xyloglucan hydrolysis. XTH proteins generally contain an N-terminal signal peptide, a GH16 catalytic domain, and a C-terminal XET_C extension. The GH16 domain contains the conserved ExDxE catalytic motif, which is required for xyloglucan transglycosylation and, in some XTH proteins, hydrolytic activity [11,12]. A putative N-glycosylation sequon is frequently located near the catalytic region and may influence protein folding, stability, or catalytic activity [11,13]. In addition, several conserved cysteine residues are commonly present in the C-terminal region and may contribute to protein structural stability [7,11]. Together, these structural features are commonly used to assess whether XTH proteins retain the canonical domain architecture. Previous studies have shown that XTHs participate in cell-wall loosening, organ elongation, plant growth and development, and stress adaptation [7,11,12,13,14,15,16,17]. Because the *XTH* gene family comprises multiple members that may differ in sequence structure, enzymatic properties, expression patterns, and biological functions, family-level identification and comparative analysis are important for understanding their potential roles in specific developmental processes [7,18,19,20,21,22,23]. *XTH* gene family analyses have been conducted in numerous plant species, including *Arabidopsis thaliana* [24], *Oryza sativa* [25], *Populus trichocarpa* [21], *Brassica rapa* [26], *Brassica oleracea* [26], *Schima superba* [18], *Beta vulgaris* [27]*,* and *Glycine max* [28]. These studies revealed considerable variation in family size, duplication patterns, conserved-domain composition, and tissue-, hormone-, and stress-responsive expression profiles, demonstrating both the evolutionary conservation and functional diversification of plant XTHs.

Tea plant germplasm exhibits high genetic diversity, and structural variation and gene presence–absence variation (PAV) are widespread among accessions. Such variation may alter gene-family size and member composition and may contribute to the genetic basis of agronomic trait differentiation [29,30]. A previous study identified *XTH* genes in tea plants and investigated their expression responses to aluminum and fluoride treatments [31]. However, previous tea XTH research has focused primarily on a single genomic background and stress-responsive expression. A systematic multi-accession comparison of the tea XTH family remains limited, leaving accession-level variation in *XTH* gene composition insufficiently characterized. Moreover, experimental evidence linking individual *CsXTH* genes to shoot internode elongation remains limited.

To clarify the conservation and accession-level variation in the *XTH* gene family and its potential relationship with shoot internode growth, we conducted a comparative analysis of *XTH* genes across nine tea genomes. Family members were systematically identified and characterized with respect to their evolutionary relationships, conserved structural features, and orthogroup-based presence–absence variation, allowing the core, variable, and accession-specific orthogroup components of the tea XTH family to be distinguished. Promoter cis-acting elements were analyzed to assess the potential regulatory characteristics of tea *XTH* genes. Tissue-expression profiles and expression comparisons between the second and third internodes were then used to identify candidate genes associated with internode development. Among these genes, *CsXTH27* was selected as a representative candidate for further sequence analysis and transient overexpression validation based on its internode-associated expression, retention of the characteristic XTH structural features, and phylogenetic position. This study establishes a multi-accession genomic framework for understanding variation in the tea *XTH* gene family and provides preliminary functional evidence supporting the involvement of *CsXTH27* in shoot internode elongation.

## 2. Results

### 2.1. Identification and Characterization of XTH Genes Across Multiple Tea Accessions

Based on conserved-domain searches, homology searches, and domain validation, 477 putative *XTH* genes were identified across the genomes of nine tea accessions, with the number of genes ranging from 42 to 66 per accession. Among them, FDDB contained the largest number of *XTH* genes, whereas LJ43 contained the fewest (Figure S1). These results indicate variation in *XTH* gene number among the examined tea genomes.

**Figure 1:**
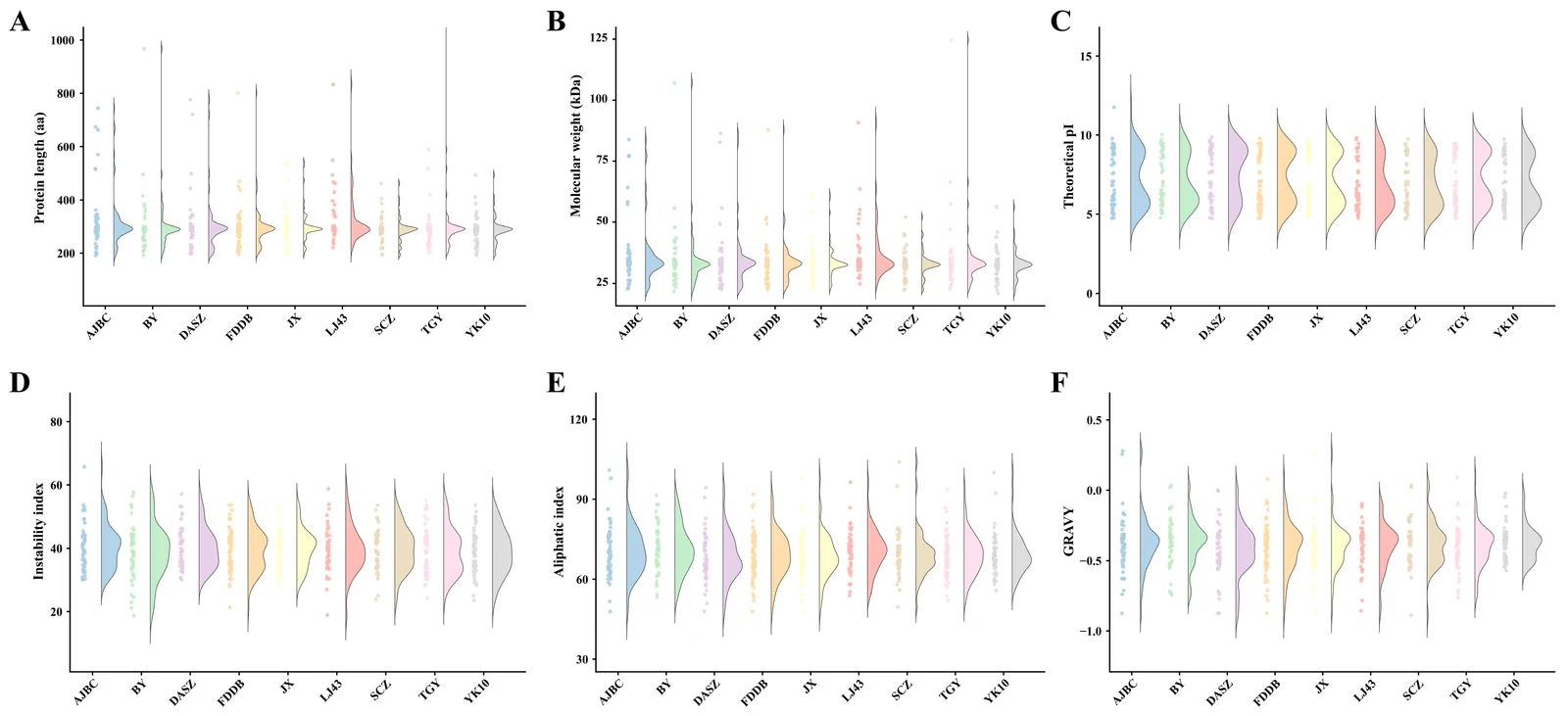
Physicochemical property distributions of XTH proteins identified in the genomes of nine tea accessions. (**A**) Protein length, (**B**) molecular weight, (**C**) theoretical isoelectric point (pI), (**D**) instability index, (**E**) aliphatic index, and (**F**) grand average of hydropathicity (GRAVY). Each dot represents an individual XTH protein, and violin plots show the distribution of each physicochemical parameter within each accession. AJBC, ‘Anji Baicha’; BY, ‘Biyun’; DASZ; FDDB, ‘Fuding Dabaicha’; JX, ‘Jinxuan’; LJ43, ‘Longjing 43’; SCZ, ‘Shuchazao’; TGY, ‘Tieguanyin’; YK10, ‘Yunkang 10’.

Further analysis of the physicochemical properties of XTH proteins showed that their overall distributions were similar among the nine accessions. Most XTH proteins were 200–400 amino acids in length, with molecular weights mainly ranging from 25 to 50 kDa. A small number of XTH proteins approached 1000 amino acids in length and approximately 125 kDa in molecular weight. The theoretical isoelectric points were mainly distributed between 5 and 10, with some values exceeding 10. Instability indices were mostly concentrated between 30 and 50, aliphatic indices generally ranged from 60 to 80, and GRAVY values were predominantly negative (Figure 1A–F). These results indicate broadly similar physicochemical properties among tea XTH proteins from the nine accessions, although a subset of proteins showed distinct sequence lengths and predicted properties.

### 2.2. Phylogenetic Relationships, Conserved Motifs, Domain Conservation, and Orthogroup-Based PAV Analysis of the Tea XTH Gene Family

**Figure 2:**
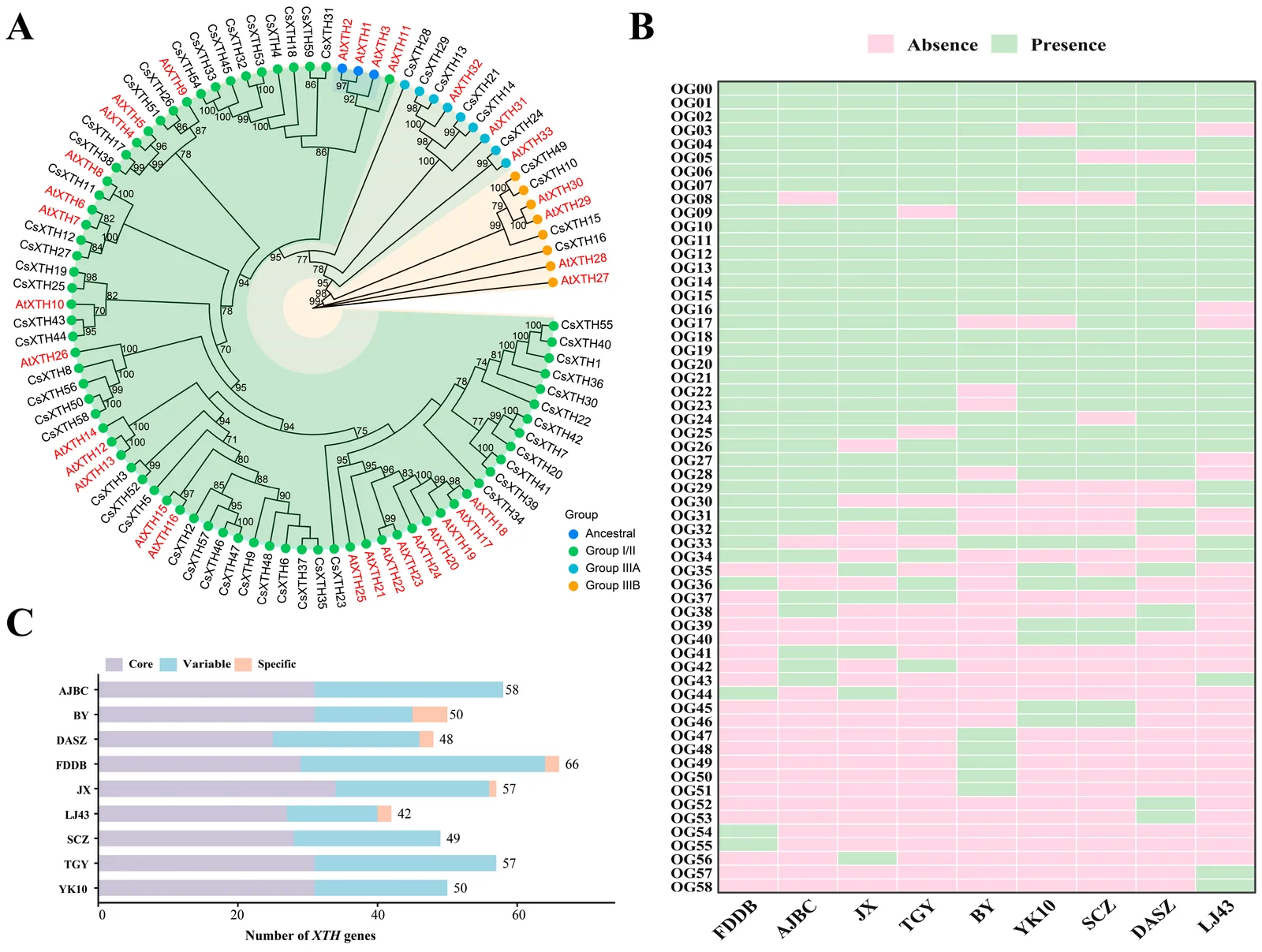
Phylogenetic relationships and orthogroup-based presence–absence variation in *XTH* genes across nine tea accessions. (**A**) Phylogenetic relationships among representative XTH proteins from *Camellia sinensis* (black) and *Arabidopsis thaliana* (red). Colored sectors and terminal symbols indicate the four major phylogenetic groups. Numbers at the nodes indicate bootstrap support values (%). (**B**) Presence–absence variation patterns of 59 XTH orthogroups across nine tea accessions. Green and pink cells indicate the presence and absence of the corresponding orthogroups, respectively. Abbreviated labels OG00–OG58 are used for readability; the corresponding original orthogroup IDs, representative *CsXTH* designations, and gene members are provided in Table S1. (**C**) Numbers of XTH genes assigned to core, variable, and accession-specific orthogroups in each tea accession. Numbers at the ends of the bars indicate the total number of XTH genes identified in each accession.

Based on the established phylogenetic classification of the XTH family [7,12,32], 59 representative tea XTH proteins and XTH proteins from *Arabidopsis thaliana* were classified into four major groups: Group I/II, Group IIIA, Group IIIB, and the Ancestral group (Figure 2A). Tea XTH proteins were distributed across all major clades and generally clustered with the corresponding Arabidopsis XTH reference proteins, supporting the phylogenetic classification of the tea XTH family.

Conserved-motif analysis of the 59 representative tea XTH proteins, together with Arabidopsis XTH proteins, showed that proteins within the same phylogenetic group generally exhibited similar motif compositions and arrangements, whereas differences were observed among groups (Figure S2). Family-wide structural analysis was subsequently performed for all 477 putative tea XTH proteins. Among them, 474 contained a detectable GH16 domain, 391 contained an XET_C domain, 450 retained the conserved ExDxE catalytic motif, and 379 retained all three characteristic features. Putative N-X-S/T glycosylation sequons were detected in 441 proteins (Table S2). Together, these results indicate that the major structural features of the tea XTH family are broadly conserved, although variation in domain and motif retention was observed among individual proteins. Orthogroup-based analysis identified 59 XTH orthogroups across the nine tea genomes (Figure 2B), abbreviated as OG00–OG58 for clarity, with original identifiers and gene members provided in Table S1. Based on their distribution among the nine genomes, the orthogroups were classified as core, variable, or accession-specific. In each accession, most XTH genes were assigned to core and variable orthogroups, whereas genes assigned to accession-specific orthogroups were less abundant (Figure 2C).

Subcellular localization analysis predicted that most tea XTH proteins were extracellular, whereas smaller subsets were predicted to localize to the cytosol, nucleus, chloroplasts, and other compartments (Figure S3).

### 2.3. Cis-Acting Element Analysis of CsXTH Promoters

**Figure 3:**
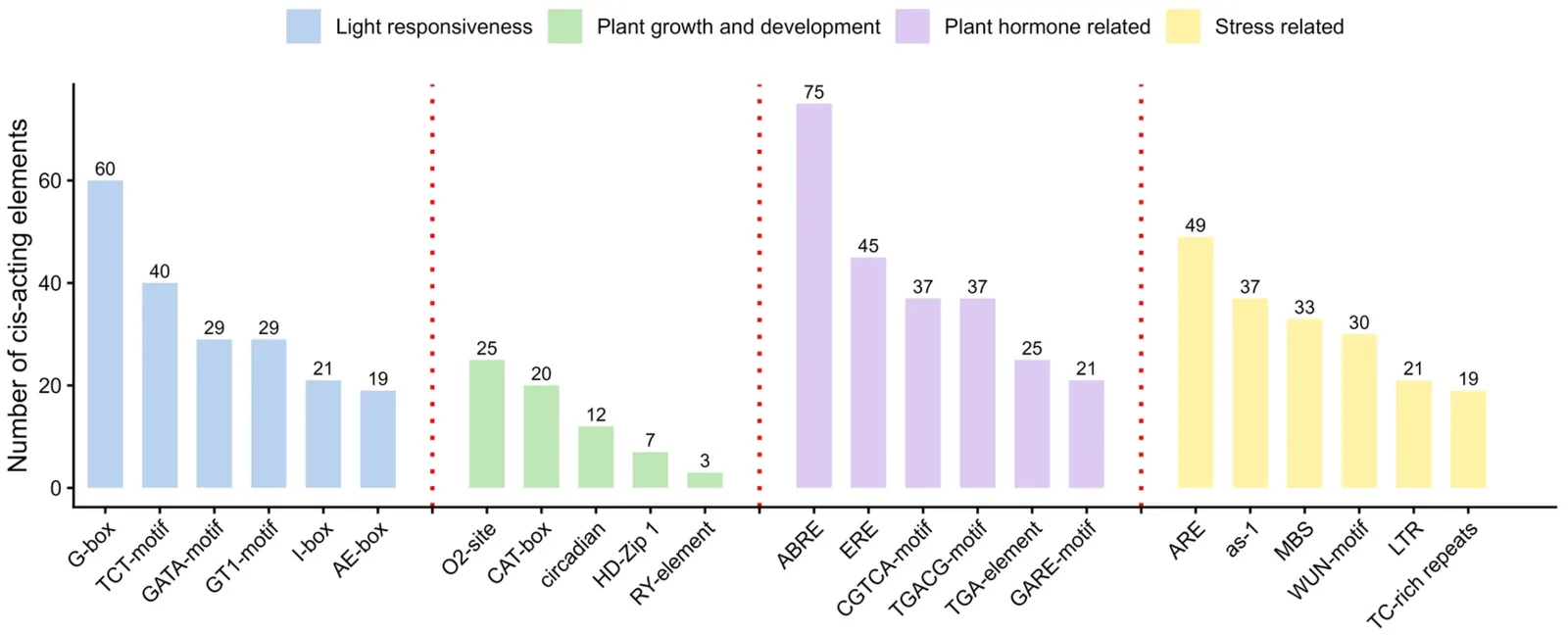
Predicted cis-acting elements in the promoter regions of *CsXTH* genes. Cis-acting elements were predicted from the 2 kb upstream regions of *CsXTH* genes in the SCZ reference genome and grouped into four functional categories: light responsiveness, plant growth and development, plant hormone-related responses, and stress-related responses. Bars represent the number of each cis-acting element, and values above the bars indicate the corresponding counts.

The predicted promoter regions of *CsXTH* genes contained numerous cis-acting elements associated with light responsiveness, plant growth and development, hormone responsiveness, and stress responsiveness (Figure 3). Among the light-responsive elements, the G-box was the most abundant, followed by the TCT-motif, GATA-motif, and GT1-motif. Among hormone-related elements, ABRE was the most abundant, followed by ERE, CGTCA-motif, TGACG-motif, TGA-element, and GARE-motif. Stress-responsive elements, including ARE, as-1, MBS, WUN-motif, LTR, and TC-rich repeats, were also frequently detected. These results suggest that *CsXTH* genes may be regulated by multiple light-, hormone-, development-, and stress-related signals.

### 2.4. Tissue and Internode Expression Patterns of CsXTH Genes

**Figure 4:**
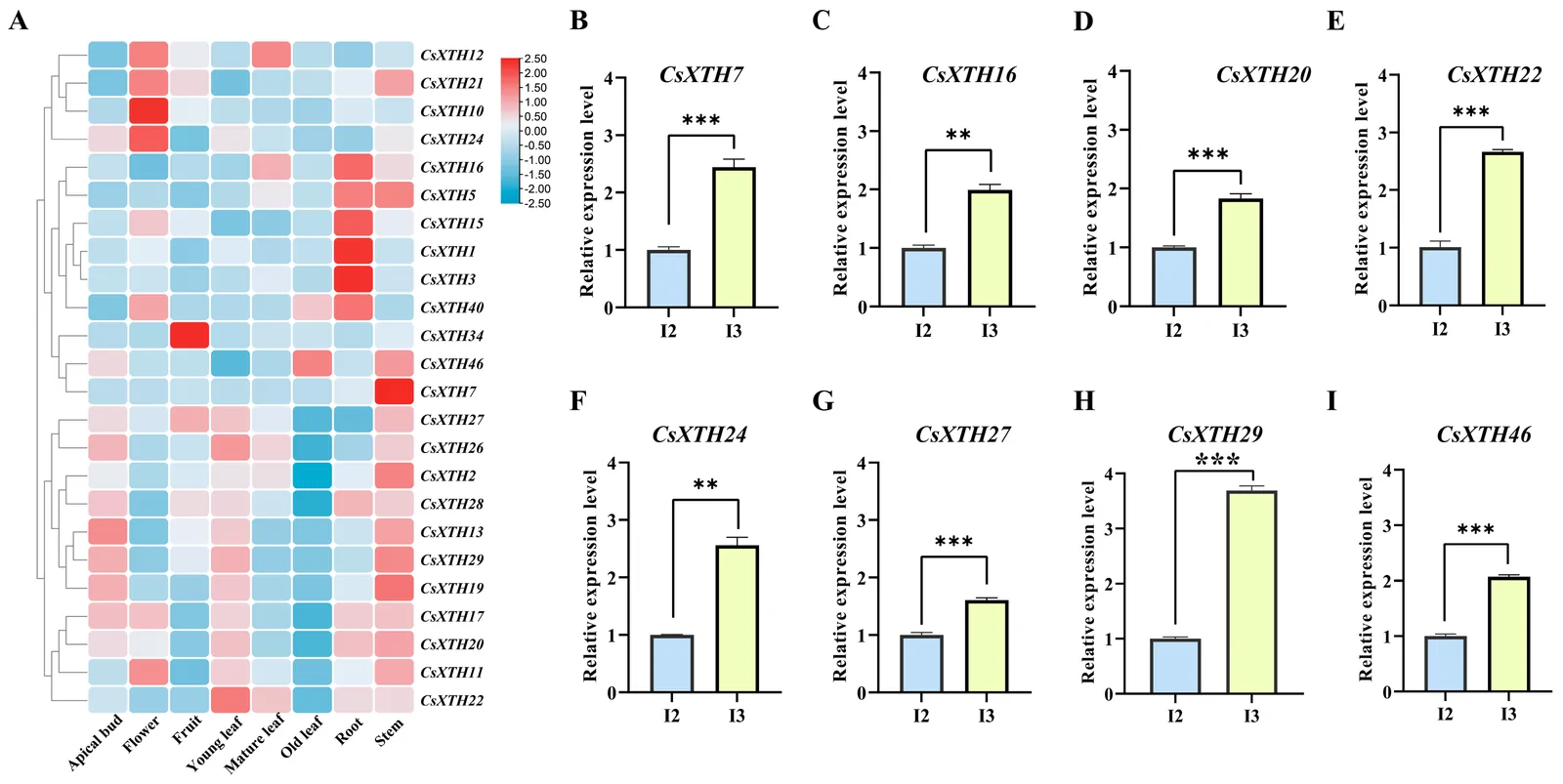
Tissue and internode expression patterns of *CsXTH* genes in tea plants. (**A**) Heatmap showing the expression profiles of *CsXTH* genes across different tissues based on the SCZ/Shuchazao v2 RNA-seq dataset, including apical bud, flower, fruit, young leaf, mature leaf, old leaf, root, and stem. Colors represent row-wise Z-scores calculated from log_2_(TPM + 1)-transformed TPM values. (**B**–**I**) RT-qPCR analysis of eight candidate *CsXTH* genes in the second internode (I2) and third internode (I3) of ‘Longjing 43’, including (**B**) *CsXTH7*, (**C**) *CsXTH16*, (**D**) *CsXTH20*, (**E**) *CsXTH22*, (**F**) *CsXTH24*, (**G**) *CsXTH27*, (**H**) *CsXTH29*, and (**I**) *CsXTH46*. Relative expression levels were normalized to *CsGAPDH*, with the expression level in I2 set to 1. Data are shown as means ± SEM of three independent biological replicates (n = 3). Asterisks indicate significant differences between I2 and I3 according to a two-tailed Student’s *t*-test: ** *p* < 0.01 and *** *p* < 0.001.

To examine the tissue-associated expression patterns of *CsXTH* genes and identify potential candidates associated with internode development, publicly available transcriptomic data from the SCZ/Shuchazao v2 dataset in TPIA [33] were analyzed. Based on their detectable expression in stems and distinct tissue-expression patterns, eight *CsXTH* genes—*CsXTH7*, *CsXTH16*, *CsXTH20*, *CsXTH22*, *CsXTH24*, *CsXTH27*, *CsXTH29*, and *CsXTH46*—were selected for further internode expression analysis (Figure 4A).

To examine their expression patterns during internode development, the relative expression levels of these eight candidate genes were compared between the second internode (I2) and third internode (I3). RT–qPCR analysis showed that all eight candidate genes were expressed at significantly higher levels in I3 than in I2 (Figure 4B–I). These results indicate that multiple *CsXTH* genes are differentially expressed between the second and third internodes.

To determine whether the differential expression of *CsXTH27* between I2 and I3 was specific to LJ43, two additional tea cultivars, BHZ and ZC108, were examined. In both cultivars, *CsXTH27* expression was significantly higher in I3 than in I2, consistent with the expression pattern observed in LJ43 (Figure S4). These results indicate that the internode-associated expression pattern of *CsXTH27* was consistently observed in all three tea cultivars examined.

### 2.5. Domain Architecture and Sequence Conservation of CsXTH27 and Representative XTH Proteins

**Figure 5:**
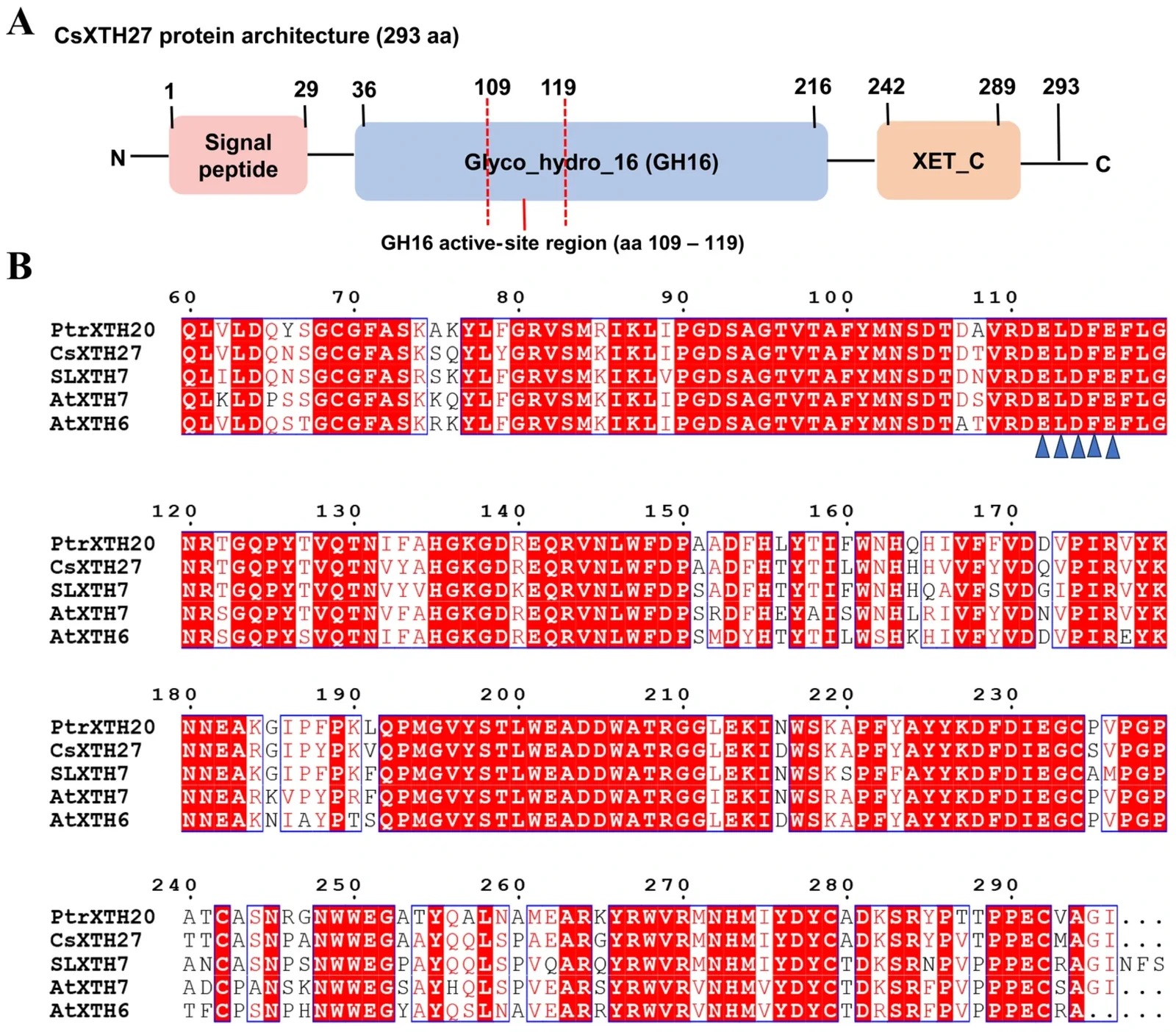
Predicted domain architecture and sequence conservation of CsXTH27 and representative XTH proteins. (**A**) Predicted domain architecture of the 293-amino-acid CsXTH27 protein. The N-terminal signal peptide (aa 1–29), Glyco_hydro_16 (GH16) domain (aa 36–216), GH16 active-site region (aa 109–119), and XET_C domain (aa 242–289) were identified using InterProScan. (**B**) Partial multiple-sequence alignment of conserved regions in CsXTH27 and representative XTH proteins, including PtrXTH20 from *Populus trichocarpa*, SlXTH7 from *Solanum lycopersicum*, and AtXTH6 and AtXTH7 from *Arabidopsis thaliana*. Identical residues are shown as white letters on a red background, whereas conserved substitutions are shown as red letters boxed in blue. Blue triangles mark the conserved ExDxE catalytic motif, corresponding to ELDFE at aa 112–116 in CsXTH27. Ptr, *P. trichocarpa*; Sl, *S. lycopersicum*; At, *A. thaliana*. Dots indicate terminal gaps in the multiple sequence alignment caused by differences in sequence length.

Among the eight candidate *CsXTH* genes, *CsXTH27* was selected as a representative candidate for further analysis based on its internode-associated expression, retention of characteristic XTH structural features, and phylogenetic proximity to AtXTH6 and AtXTH7. The full-length coding sequence of *CsXTH27* was amplified from stem cDNA of the tea cultivar ‘Longjing 43’ (LJ43), and its identity was confirmed by Sanger sequencing. The verified coding sequence encoded a protein of 293 amino acids. InterProScan analysis predicted that the CsXTH27 protein contained an N-terminal signal peptide spanning residues 1–29, a Glyco_hydro_16 (GH16) domain spanning residues 36–216, and an XET_C domain spanning residues 242–289 (Figure 5A). A predicted GH16 active-site region spanning residues 109–119 contained the conserved ExDxE catalytic motif, corresponding to ELDFE at residues 112–116 in CsXTH27.

Comparison of CsXTH27 with PtrXTH20, SlXTH7, AtXTH6, and AtXTH7 revealed extensive sequence conservation within the GH16 catalytic region and the C-terminal XET_C domain (Figure 5B), with the ExDxE catalytic motif strictly conserved among all five proteins. Several additional residues surrounding the catalytic region and within the C-terminal region were also identical or highly conserved. Collectively, these sequence features support the annotation of CsXTH27 as a structurally conserved XTH protein. However, conservation of the characteristic domains and catalytic motif alone does not demonstrate XTH enzymatic activity.

### 2.6. Transient Overexpression of CsXTH27 Increases Internode Length in Tea Shoots

**Figure 6:**
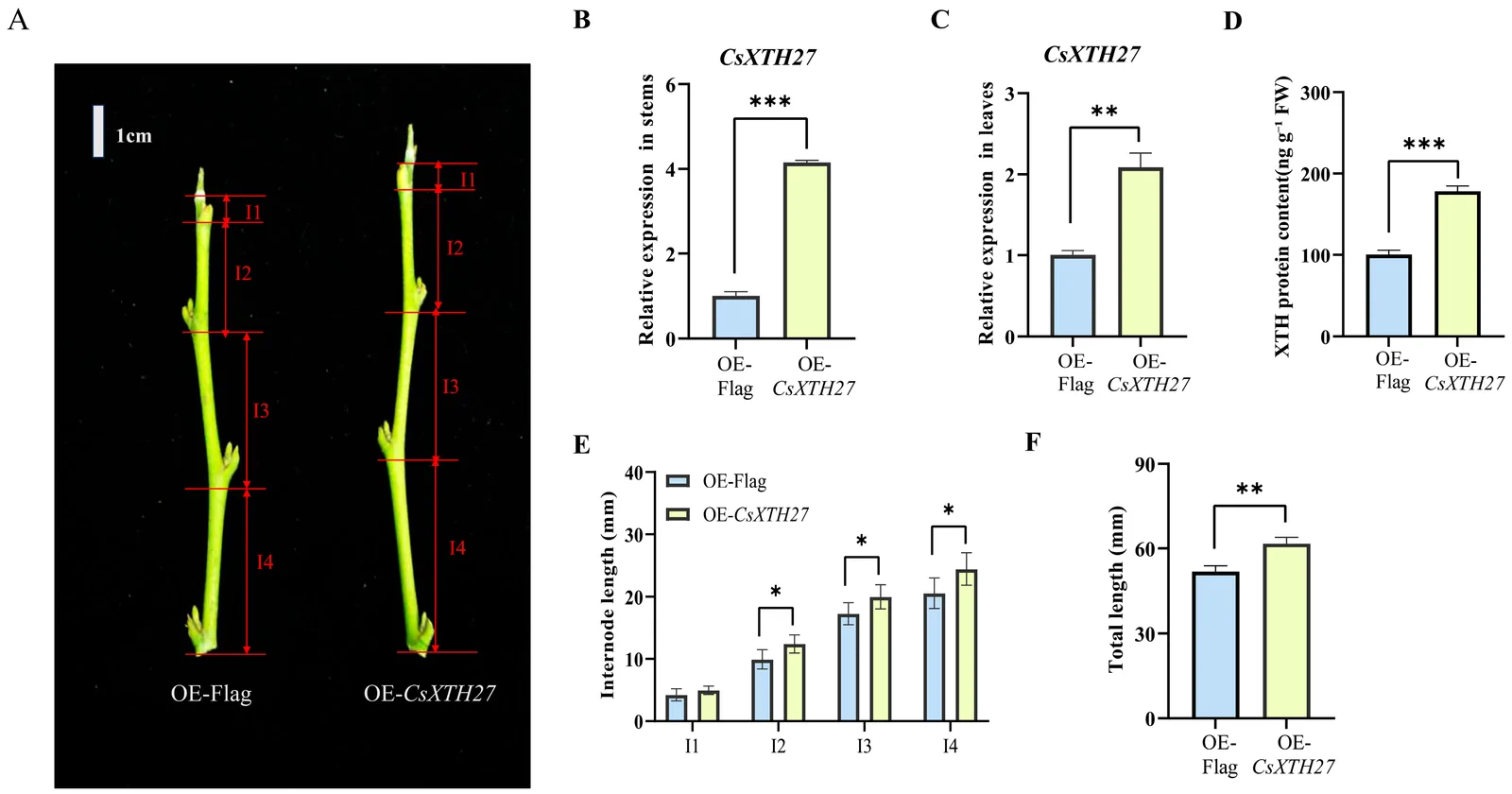
Effects of transient *CsXTH27* overexpression on gene expression, XTH protein content, and internode length in young shoots of the tea cultivar ‘Longjing 43’ (LJ43). (**A**) Representative phenotypes of LJ43 shoots transiently transformed with the empty 35S::Flag vector (OE-Flag) or the 35S::*CsXTH27*::Flag construct (OE-*CsXTH27*). Red horizontal lines and double-headed arrows indicate the boundaries and lengths of internodes I1–I4. Scale bar = 1 cm. (**B**,**C**) Relative expression levels of *CsXTH27* in the stems (**B**) and leaves (**C**) of OE-Flag and OE-*CsXTH27* shoots. Expression levels were normalized to *CsGAPDH*, with the expression level in OE-Flag set to 1 for each tissue. (**D**) XTH protein content in the stems of OE-Flag and OE-*CsXTH27* shoots, expressed as ng g^−1^ fresh weight (FW). (**E**) Lengths of individual internodes I1–I4 in OE-Flag and OE-*CsXTH27* shoots. (**F**) Total length of internodes I1–I4. Data are presented as means ± SEM. The RT-qPCR and XTH protein-content analyses in panels (**B**–**D**) were based on three independent biological replicates (n = 3), whereas the internode-length measurements in panels (**E**,**F**) were based on nine independently transformed shoots per treatment (n = 9). Asterisks indicate significant differences between OE-Flag and OE-*CsXTH27* according to a two-tailed Student’s *t*-test: * *p* < 0.05, ** *p* < 0.01, and *** *p* < 0.001.

To evaluate the potential role of *CsXTH27* in internode elongation, *CsXTH27* was transiently overexpressed in young shoots of the tea cultivar ‘Longjing 43’ (LJ43). Compared with the empty-vector control (OE-Flag), OE-*CsXTH27* shoots appeared to have longer I2, I3, and I4 internodes (Figure 6A). RT–qPCR analysis confirmed that *CsXTH27* expression was significantly higher in both the stems and leaves of OE-*CsXTH27* shoots than in OE-Flag controls (Figure 6B,C). XTH protein content in stems was also significantly higher in OE-*CsXTH27* shoots than in OE-Flag controls (Figure 6D). Quantitative analysis showed that the I2, I3, and I4 internodes were significantly longer in OE-*CsXTH27* shoots, whereas no significant difference was observed for I1 (Figure 6E). Accordingly, the total length of I1–I4 was significantly greater in OE-*CsXTH27* shoots than in OE-Flag controls (Figure 6F). Histological observations further showed an apparent tendency toward larger pith parenchyma cell profiles in OE-*CsXTH27* internodes than in OE-Flag controls (Figure S5). These results provide preliminary evidence that transient overexpression of *CsXTH27* increases internode length and suggest that enhanced pith-cell expansion may contribute to this phenotype.

## 3. Discussion

A previous study identified XTH genes in tea plants using a single genomic background and mainly investigated their expression responses to aluminum and fluoride treatments [31]. By comparing nine tea genomes, the present study provides a broader view of the conservation and variation in the tea XTH family. Most of the identified proteins retained the characteristic GH16 and XET_C domains and the conserved ExDxE catalytic motif, indicating that the principal structural framework of the tea XTH family is broadly conserved. In contrast, variation was evident in gene number and in the distribution of variable and accession-specific orthogroups among the examined genomes (Figure 2B,C; Table S2). Extensive structural and presence–absence variation has also been reported among tea accessions [29,30]. This pattern suggests that the tea XTH family comprises a conserved component that may support fundamental cell-wall-related functions and a more variable component that may contribute to functional diversification among accessions. However, the present analysis does not directly associate individual variable orthogroups with differences in shoot architecture or internode length. In addition, proteins lacking one or more canonical structural feature may represent divergent XTH-like members, incomplete gene models, or annotation artifacts, and their biochemical properties cannot be inferred from sequence analysis alone. These findings extend tea XTH research from a single-reference perspective to a multi-genome framework. However, genuine family variation should be distinguished from apparent differences arising from variation in genome assembly and annotation quality [34].

Expression profiling further revealed an association between XTH family members and internode development. Eight candidate *CsXTH* genes selected from the tissue-expression dataset were expressed at significantly higher levels in I3 than in I2 in ‘Longjing 43’ (Figure 4A–I), suggesting that internode development may involve multiple XTH members rather than a single gene. Developmental-stage-dependent expression of *XTH* genes has also been reported in other plant species [35,36,37], consistent with the involvement of XTH proteins in developmentally regulated cell-wall remodeling. However, because I2 and I3 also differ in developmental status and tissue maturity, these expression differences should be interpreted as associations with internode development rather than direct evidence that the corresponding genes determine internode length. Among these genes, *CsXTH27* was selected as a representative candidate for further analysis rather than the only relevant family member, based on its internode-associated expression, conserved XTH protein features, and phylogenetic position. The same I3-preferential expression pattern of *CsXTH27* was subsequently observed in ‘Baihaozao’ and ‘Zhongcha 108’ (Figure S4), supporting the consistency of this expression pattern across the three cultivars examined. The CsXTH27 protein retained an N-terminal signal peptide, the GH16 and XET_C domains, and the conserved ExDxE catalytic motif and was phylogenetically related to AtXTH6 and AtXTH7 (Figure 2A and Figure 5A,B). The corresponding *Arabidopsis* genes, *AtXTH6* and *AtXTH7,* exhibit distinct developmental expression patterns [35]. However, this phylogenetic relationship and expression-related context do not establish functional equivalence among these proteins. In tea shoots, transient overexpression of *CsXTH27* significantly increased the lengths of I2, I3, and I4, as well as the total length of I1–I4, whereas I1 length was not significantly affected (Figure 6E,F). This pattern suggests that the effect of transient CsXTH27 overexpression may vary with internode position or developmental stage. The consistency between its internode-associated expression pattern and overexpression phenotype provides preliminary functional evidence supporting the involvement of *CsXTH27* in shoot internode elongation.

The present evidence supports the involvement of *CsXTH27* in internode elongation, although the precise cellular and biochemical mechanisms remain to be fully resolved. Histological observations showed an apparent tendency toward larger pith parenchyma cell profiles in OE-*CsXTH27* internodes than in OE-Flag controls (Figure S5), suggesting that enhanced cell expansion may contribute to the increased internode length. Quantitative measurements of cell size and cell number are therefore required to distinguish the relative contributions of cell expansion and cell proliferation to this phenotype. In addition, XTH protein content was significantly higher in OE-*CsXTH27* stems than in OE-Flag controls (Figure 6D), providing evidence consistent with increased XTH protein accumulation. However, the ELISA measurement reflects protein abundance and does not directly demonstrate xyloglucan endotransglucosylase or hydrolase activity. Direct enzymatic assays will therefore be necessary to determine whether the increased XTH protein accumulation is accompanied by altered xyloglucan endotransglucosylase or hydrolase activity [38]. Stable overexpression and loss-of-function studies would further clarify the role of *CsXTH27* in internode growth. Overall, this study extends previous tea XTH research by revealing a broadly conserved structural framework together with accession-level variation in XTH family composition across nine tea genomes and by providing preliminary functional evidence linking *CsXTH27* to shoot internode elongation.

## 4. Materials and Methods

### 4.1. Plant Materials and Genomic Resources

Tea plants of ‘Longjing 43’ (LJ43), ‘Baihaozao’ (BHZ), and ‘Zhongcha 108’ (ZC108) were grown under the same cultivation conditions in the experimental field of the Tea Research Institute, Chinese Academy of Agricultural Sciences, Hangzhou, China. Young shoots with comparable growth status were selected from each cultivar. The second and third internodes (I2 and I3) were collected from LJ43 for expression analysis of the selected *CsXTH* genes and from BHZ and ZC108 for cross-cultivar validation of the I2–I3 expression pattern of *CsXTH27*. Young LJ43 shoots with comparable growth status and initial internode lengths were selected for the transient overexpression assays. Three independent biological replicates were included in the expression analyses. Nine independently transformed LJ43 shoots per treatment were used for internode-length measurements in the transient overexpression assay.

Genome assemblies and protein annotation datasets from nine tea accessions—DASZ, AJBC, BY, FDDB, JX, SCZ, LJ43, TGY, and YK10—were used for XTH family identification and comparative genomic analysis [30,39,40,41,42,43,44,45]. These accessions were selected based on the availability of publicly accessible genome assemblies and matched protein annotation datasets suitable for comparative gene-family analysis. Specifically, the AJBC dataset was obtained from Tariq et al. [30], whereas the FDDB and JX datasets were obtained from Chen et al. [41]. The genome assemblies and corresponding protein annotation datasets of DASZ, BY, SCZ, LJ43, TGY, and YK10 were downloaded from the Tea Plant Information Archive (TPIA) [33]. The genome assembly and annotation versions specified in the corresponding source datasets were used throughout this study.

### 4.2. Identification of XTH Gene Family Members in Tea Plants

XTH family members were identified using a combined strategy based on conserved protein domains and sequence homology [17]. The HMM profiles of the GH16 domain (PF00722) and XET_C domain (PF06955) obtained from the Pfam database were used to search the predicted protein sequences of each tea accession. The candidate sequences were further validated using the NCBI Conserved Domain Database (CDD), and previously reported XTH protein sequences from *Arabidopsis thaliana* [35] were used as queries for additional BLASTp searches using the NCBI Protein BLAST web server (accessed on 18 November 2025). Sequences were retained when they were supported by at least one domain-based search together with detectable homology to previously characterized plant XTH proteins. Sequences lacking both recognizable XTH-related domains and convincing sequence-homology support were excluded. To reduce redundancy, sequences showing greater than 99% identity were aligned, and only one representative sequence was retained for subsequent analyses.

### 4.3. Prediction of Physicochemical Properties and Subcellular Localization

The physicochemical properties of XTH proteins, including protein length, molecular weight (MW), theoretical isoelectric point (pI), instability index, aliphatic index, and grand average of hydropathicity (GRAVY), were calculated using the Physicochemical Properties module in TBtools-II v2.420 [46]. Subcellular localization was predicted using WoLF PSORT (https://wolfpsort.hgc.jp, accessed on 24 December 2025).

### 4.4. Phylogenetic and Cis-Acting Element Analyses

Multiple sequence alignment of XTH protein sequences was performed using ClustalW (https://www.genome.jp/tools-bin/clustalw, accessed on 9 December 2025). A maximum-likelihood (ML) phylogenetic tree was constructed using IQ-TREE [47], with the best-fit amino acid substitution model selected automatically. Branch support was evaluated using 1000 bootstrap replicates [48]. For cis-acting element analysis, the SCZ reference genome was used as a unified reference because the tissue-expression dataset analyzed in this study was also based on SCZ, thereby ensuring consistency in gene identifiers between the promoter and expression analyses. The 2 kb upstream sequences of individual *CsXTH* genes were extracted from the SCZ reference genome using TBtools. Cis-acting elements were predicted using PlantCARE (https://bioinformatics.psb.ugent.be/webtools/plantcare/html/, accessed on 27 December 2025) [49].

### 4.5. Presence–Absence Variation Analysis of XTH Genes

To evaluate the presence–absence variation (PAV) of the *XTH* gene family among the nine tea accessions [50], the XTH protein sequences identified from each accession were clustered into orthogroups using OrthoFinder v2.3.8 [51]. A binary PAV matrix was constructed at the orthogroup level, with an orthogroup scored as present in an accession when at least one corresponding *XTH* gene was detected. Orthogroups present in all nine accessions were classified as core, those present in two to eight accessions as variable, and those detected in only one accession as accession-specific. The PAV matrix was visualized as a heatmap in R version 4.5.1 (R Foundation for Statistical Computing, Vienna, Austria).

### 4.6. Transcriptomic Expression Profiling of CsXTH Genes

Transcriptomic expression data for tea *XTH* genes in different tissues, represented as transcripts per million (TPM), were obtained from the SCZ/Shuchazao v2 dataset in the Tea Plant Information Archive (TPIA) [33]. TPM values were log_2_-transformed using log_2_(TPM + 1) and subsequently standardized for each gene by row-wise Z-score normalization to compare relative expression patterns across tissues. A heatmap with hierarchical clustering was generated using TBtools to visualize the expression patterns of *CsXTH* genes across eight tissues, including apical buds, flowers, fruits, young leaves, mature leaves, old leaves, roots, and stems.

### 4.7. Protein Domain and Sequence Analyses

The retained XTH protein sequences were further examined based on the Pfam and CDD results to record the presence and positions of the GH16 and XET_C domains. The conserved ExDxE catalytic motif, putative N-glycosylation sequons matching the N-X-S/T pattern, where X represents any amino acid other than proline, and cysteine residues within the predicted XET_C domain were identified by sequence-pattern analysis. Conserved motifs were identified using MEME.

*CsXTH27* corresponds to the gene model CSS0033400 in the ‘Shuchazao’ reference genome. The full-length coding sequence of *CsXTH27* was amplified from stem cDNA of the tea cultivar ‘Longjing 43’ and verified by Sanger sequencing. The deduced amino acid sequence was analyzed using InterProScan version 5.78–109.0 [52] to identify the signal peptide, GH16 domain, active-site region, and XET_C domain. The CsXTH27 amino acid sequence was aligned with representative XTH proteins, including PtrXTH20 from *Populus trichocarpa* [21], SlXTH7 from *Solanum lycopersicum* [53], and AtXTH6 and AtXTH7 from *Arabidopsis thaliana* [32,54]. Multiple sequence alignment was performed using the online ClustalW tool with default parameters. The alignment results were visualized using ESPript 3.0 (https://espript.ibcp.fr/ESPript/ESPript/, accessed on 20 April 2026) [55].

### 4.8. RNA Extraction, cDNA Synthesis, and RT-qPCR Analysis

The second internode (I2) and third internode (I3) tissues were collected from the tea cultivar ‘Longjing 43’ (LJ43) for expression analysis of the selected *CsXTH* genes. To validate the expression pattern of *CsXTH27* across cultivars, I2 and I3 were also collected from ‘Baihaozao’ (BHZ) and ‘Zhongcha 108’ (ZC108). For validation of transient *CsXTH27* overexpression, stem and leaf tissues were collected separately from OE-Flag and OE-CsXTH27 shoots. All samples were immediately frozen in liquid nitrogen and stored at −80 °C until RNA extraction. Total RNA was extracted using the RNA Simple Total RNA Kit (TIANGEN, Beijing, China) according to the manufacturer’s instructions. RNA quality and concentration were determined, and equal amounts of total RNA were used for first-strand cDNA synthesis using a reverse transcription kit (AG, Changsha, Hunan, China).

RT-qPCR analysis was performed using a SYBR Green Pro Taq HS fluorescent dye kit (AG, Changsha, Hunan, China) on a LightCycler^®^ 480 II real-time PCR system (Roche Diagnostics, Mannheim, Germany). All primers used for RT-qPCR in this study are listed in Table S3. *CsGAPDH* was used as the internal reference gene to normalize target-gene expression and correct for sample-to-sample variation. The RT-qPCR program was as follows: 95 °C for 30 s, followed by 45 cycles of 95 °C for 10 s and 60 °C for 30 s. A melting-curve analysis from 60 to 95 °C was subsequently performed to confirm amplification specificity. Relative gene expression levels were calculated using the 2^−ΔΔCt^ method. Three independent biological replicates were analyzed for each sample.

### 4.9. Transient Overexpression of CsXTH27 in Tea Plants

The full-length coding sequence of *CsXTH27* was amplified from stem-derived cDNA of the tea cultivar ‘Longjing 43’ (LJ43). The primers used for *CsXTH27* cloning and overexpression vector construction are listed in Table S4. The amplified product was cloned into the 35S::Flag vector to generate the 35S::*CsXTH27*::Flag construct, which was then introduced into *Agrobacterium tumefaciens* GV3101 (pSoup-p19). The empty 35S::Flag vector served as the control. Young shoots of the tea cultivar LJ43 were vacuum-infiltrated with the corresponding *Agrobacterium* suspensions and maintained for 5 d under greenhouse conditions. Phenotypes were photographed, and internode lengths were measured using a vernier caliper. Stem and leaf tissues were collected separately for RT–qPCR validation of *CsXTH27* overexpression. Stem tissues were additionally collected for XTH protein-content analysis. All samples used for molecular and protein analyses were immediately frozen in liquid nitrogen and stored at −80 °C until use. Internode segments collected from corresponding positions were used for histological observation. Nine independently transformed shoots were used for phenotypic measurements per treatment (n = 9). Three independent biological replicates were used for RT-qPCR and XTH protein-content analyses.

### 4.10. Measurement of XTH Protein Content

Stem tissues were collected from OE-Flag and OE-CsXTH27 shoots and homogenized in pre-cooled phosphate-buffered saline (PBS; 0.01 M, pH 7.4) at a tissue-to-buffer ratio of 1:9 (*w*/*v*). The homogenates were centrifuged at 5000× *g* for 10 min under refrigerated conditions, and the supernatants were collected for analysis. XTH protein content was measured using a Plant Xyloglucan Endotransglucosylase/Hydrolase (XTH) ELISA Kit (Hangzhou Chuangshi Biotechnology Co., Ltd., Hangzhou, China; Cat. No. CS-172002-96T) according to the manufacturer’s instructions. Absorbance was measured at 450 nm, and XTH concentrations were calculated using a standard curve generated with standards ranging from 0 to 200 ng mL^−1^. The calculated concentrations were corrected for the fivefold sample dilution and normalized to tissue fresh weight. Three independent biological replicates were analyzed for each treatment.

### 4.11. Histological Observation of Internode Tissues

Internode segments collected from corresponding positions in OE-Flag and OE-CsXTH27 shoots were fixed and subjected to paraffin embedding and histological sectioning. Transverse and longitudinal sections were prepared and stained using standard histological procedures. Sections were digitally scanned, and the pith regions were examined at the same magnification to compare pith parenchyma cell morphology between the two treatments. Three independent biological replicates were examined for each treatment.

### 4.12. Data Analysis

Statistical analyses were performed using IBM SPSS Statistics 27. Data are presented as means ± standard error of the mean (SEM). Comparisons between two groups were conducted using a two-tailed Student’s *t*-test, whereas comparisons among multiple groups were performed using one-way analysis of variance (ANOVA) followed by Tukey’s multiple-comparison test. Figures were generated using GraphPad Prism 9, TBtools-II v2.420, and R version 4.5.1 (R Foundation for Statistical Computing, Vienna, Austria). Statistical significance was defined as *p* < 0.05. Asterisks indicate significant differences: * *p* < 0.05, ** *p* < 0.01, and *** *p* < 0.001. Unless otherwise specified, molecular and protein analyses were based on three independent biological replicates, whereas internode-length measurements were based on nine independently transformed shoots per treatment.

## 5. Conclusions

This study characterized the *XTH* gene family across the genomes of nine tea accessions and identified a total of 477 putative *XTH* genes. Phylogenetic, conserved-motif, protein-domain, and orthogroup-based PAV analyses revealed both structural conservation and accession-level variation within the tea *XTH* gene family. Tissue-expression profiling and internode expression analysis identified several *CsXTH* genes potentially associated with internode development. Among these genes, *CsXTH27* showed consistently higher expression in I3 than in I2 in LJ43, BHZ, and ZC108 and encoded a protein retaining the characteristic structural features of canonical XTH proteins. Transient overexpression of *CsXTH27* significantly increased the lengths of I2, I3, and I4 and the total length of I1–I4. This phenotype was accompanied by increased XTH protein content in stems and an apparent tendency toward larger pith parenchyma cell profiles. These findings establish a comparative genomic framework for the tea *XTH* gene family and provide preliminary functional evidence supporting the involvement of *CsXTH27* in shoot internode elongation. Further quantitative cytological analyses, direct enzymatic assays, and stable genetic validation are required to clarify the cellular and molecular mechanisms underlying the *CsXTH27*-associated phenotype.

## Data Availability

The original contributions presented in this study are included in the article/Supplementary Materials. Further inquiries can be directed to the corresponding authors.

## Supplementary Materials

The following supporting information can be downloaded at: https://www.mdpi.com/article/10.3390/ijms27167388/s1.
