# Supplementary material for: Comparative Genomics of the Tea XTH Gene Family Reveals the Involvement of CsXTH27 in Internode Elongation
Source: Int J Mol Sci. 2026 Aug 18;27(16):7388. doi: 10.3390/ijms27167388 (PMC13513827; doi:10.3390/ijms27167388)
Supplement: Supplementary file 1 [file ijms-27-07388-s001.zip › Supplemental Figure Legends.pdf]

Supplemental Figure Legends

Figure S1. Distribution of *XTH* gene numbers among nine tea accessions. The numbers of putative *XTH* genes identified in the genomes of DASZ, AJBC, BY, FDDB, JX, SCZ, LJ43, TGY, and YK10 are shown.

Figure S2. Conserved motif composition of XTH proteins from tea plants and *Arabidopsis thaliana.* A total of 59 representative tea XTH proteins, with one representative selected from each orthogroup, together with XTH proteins from *A. thaliana*, were analyzed using MEME. Motifs 1–6 are represented by boxes of different colors. Protein names and the phylogenetic tree are shown on the left.

Figure S3. Distribution of predicted subcellular localization of XTH proteins across nine tea accessions. Subcellular localization of the identified XTH proteins was predicted using WoLF PSORT. The predicted localization categories include the extracellular space, cytosol, nucleus, chloroplast, and other cellular compartments.

Figure S4. Relative expression of *CsXTH27* in the second and third internodes of two additional tea cultivars. Relative expression levels of *CsXTH27* in the second internode (I2) and third internode (I3) of (A) 'Baihaozao' (BHZ) and (B) 'Zhongcha 108' (ZC108). Expression levels were normalized to *CsGAPDH*, with the expression level in I2 set to 1. Data are presented as means ± SEM of three independent biological replicates (n = 3). Asterisks indicate significant differences between I2 and I3 according to a two-tailed Student's *t*-test: ***$p < 0.001$.

Figure S5. Histological comparison of stem tissues between OE-Flag and OE-*CsXTH27* tea shoots. Representative transverse sections of stems from (A) OE-Flag and (B) OE-*CsXTH27* shoots and longitudinal sections of stems from (C) OE-Flag and (D) OE-*CsXTH27* shoots. Black dashed outlines indicate the pith regions used for morphological comparison. Compared with the OE-Flag control, pith parenchyma cells in OE-*CsXTH27* stems showed an apparent tendency toward larger cellular profiles. Scale bars are indicated in each image.
